# Gender differences in lung cancer epidemiology – do Austrian male lung cancer patients still die earlier in life?

**DOI:** 10.3389/fpubh.2023.1099165

**Published:** 2023-04-25

**Authors:** Richard Felsinger, Ursula Kunze, Ernest Groman

**Affiliations:** Department of Social and Preventive Medicine, Center for Public Health, Medical University of Vienna, Vienna, Austria

**Keywords:** mean age of death, lung cancer, cancer epidemiology, public health, smoking, cigarettes, tobacco, gender differences

## Abstract

**Objective:**

Previous analyses reported an unexpected decline of mean age of death of Austrian male lung cancer patients until 1996 and a subsequent turnaround of this epidemiological trend after the mid-1990s until 2007. In light of ongoing changes in smoking behavior of men and women, this study aims to investigate the development of mean age of death from lung cancer in Austria during the past three decades.

**Materials and methods:**

This study used data about the annual mean age of death from lung cancer, including malignant neoplasm of trachea, bronchus and lung, between 1992 and 2021 obtained from Statistics Austria, Federal Institution under Public Law. One-way analysis of variance (ANOVA) and independent samples *t*-tests were applied to explore any significant differences of mean values in the course of time as well as between men and women.

**Results:**

Overall, mean age of death of male lung cancer patients increased consistently throughout the observed time periods, whereas women did not show any statistically significant change in the last decades.

**Conclusion:**

Possible reasons for the reported epidemiological development are discussed in this article. Research and Public Health measures should increasingly focus on smoking behaviors of female adolescents.

## 1. Introduction

**Figure 1:**
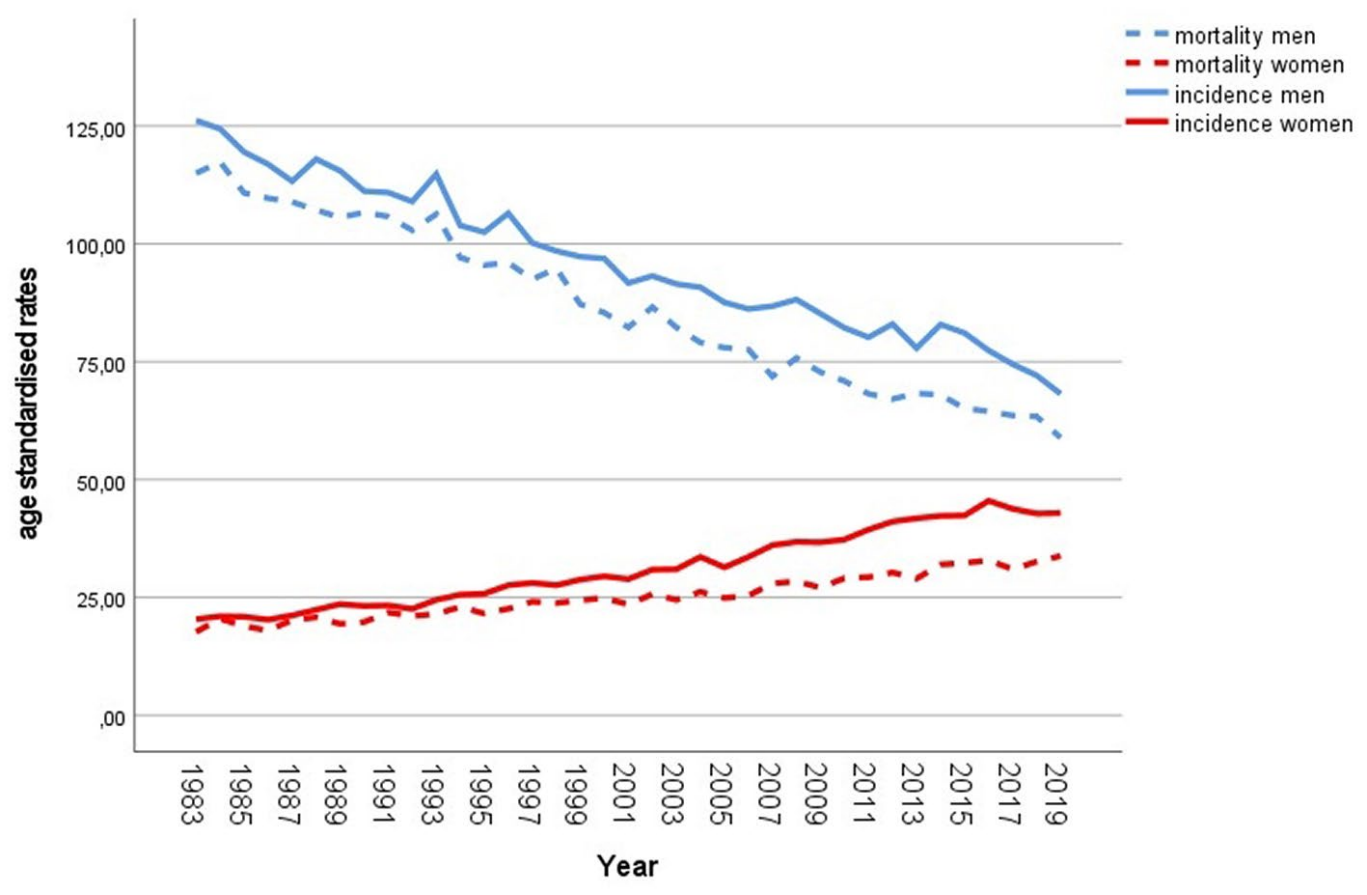
Incidence and mortality of lung cancer in Austria since 1983. Age standardized rates per 100,000 population, European Standard Population 2013. Source: Statistics Austria (2–4).

Lung cancer is the second most common cancer type in Austrian men and women (1). The Austrian National Cancer Registry, maintained by Statistics Austria, reported 2.770 men and 2.061 women newly diagnosed with malignant neoplasm of trachea, bronchus and lung (ICD 10—C33, 34) in 2019 (2). Incidence and mortality rates of men and women developed differently over the past decades (see Figure 1). Austrian men showed a large incidence and mortality decline, whereas the female rates increased constantly (3). In 2019, the age standardized mortality rate per 100,000 persons was 59 for men and 34 for women (4). The overall 5-year relative survival rate increased between the period 2000–2004 and 2010–2014 from 15% to 20%. For the last mentioned period the reported average 5-year relative survival for men and women was 17.4% and 24.5%, respectively (3).

In the early 2000s, epidemiological findings suggested that, despite increasing life expectancy and declining lung cancer incidence and mortality in men, the mean age of death of Austrian male lung cancer patients had been decreasing until 1996 (5). Consequently, Borsoi et al. reported an increase of mean age of death after the mid-1990s, but still accounted for a difference between men and women of about half a year in 2007 (6). Despite the continuing increase of life expectancy in Austria, the existing gender differences in lung cancer incidence, mortality and smoking behavior and the advances made over the past decades in the management of non-small cell lung cancer (NSCLC) (7) as well as small cell lung cancer (SCLC) (8), recent reports about mean age of death from lung cancer in Austria are missing. This study aims to provide an update on the development of the mean age of death of Austrian male and female lung cancer patients.

## 2. Materials and methods

Data about the annual mean age of death from lung cancer, including malignant neoplasm of trachea, bronchus and lung (ICD 10—C33, 34), was available upon request from Statistics Austria, Federal Institution under Public Law. The calculation of the exact empirical mean age of death is based on the difference between date of birth and date of death in days. Contrary to the life expectancy at birth, which is an age standardized measured value, the exact empirical mean age of death reflects the mean of the distribution of the actual deaths from lung cancer (9). Data was divided into three groups, comprising the following three time periods: 1992–2001, 2002–2011 and 2012–2021. In order to explore any statistically significant differences of the mean values between the three groups a one-way analysis of variance (ANOVA) was applied. Pair-wise comparisons were performed using *post-hoc* tests. All analyses were done for men and women separately. Independent samples *t*-tests were performed to explore gender differences. *p*-values ≤0.05 were considered to be statistically significant. IBM SPSS Statistics 28.0 was utilized to perform the statistical analyses.

The assumptions for ANOVA (normal distribution of data and homogeneity of variances in all groups) were tested. Normal distribution of data in all groups was visually verified using scatter plots (Supplementary material). The assumption of homogeneity of variances was assessed by applying Levene’s test. The according *p*-values of the male and female analysis were less than 0.05 (0.002 and 0.021) indicating a violation of this assumption. Hence, Welch’s ANOVA and Games-Howell *post-hoc* tests, which are more robust in case of unequal variances, were performed.

## 3. Results

Table 1 shows the number of deaths from lung cancer as well as the mean age of death of male and female Austrian lung cancer patients in each time period, the standard deviation, the 95% confidence interval and the *p*-value of the Welch ANOVA. The results indicate statistically significant differences of the mean values between the three groups in men and women. According to the Games-Howell *post-hoc* tests (Table 2), mean age of death of female lung cancer patients decreased significantly by 0.5 years between the periods 1992–2001 and 2002–2011 and increased by 0.7 years between 2002–2011 and 2012–2021. The difference between the periods 1992–2001 and 2012–2021 is statistically not significant (*p* = 0.680).

**Table 1:** Descriptive statistics and ANOVA.

	Period	Years	Deaths	Mean	SD	95% CI	*p*-value
Male	1992–2001	10	23471	68.12	0.131656	68.03; 68.21	<0.001
	2002–2011	10	23448	69.00	0.290593	68.79; 69.21	
	2012–2021	10	23991	70.72	0.608824	70.28; 71.16	
Female	1992–2001	10	8904	70.43	0.41379	70.13; 70.73	0.009
	2002–2011	10	11321	69.93	0.340098	69.69; 70.17	
	2012–2021	10	15178	70.66	0.750111	70.12; 71.20	

Number of included years, number of deaths from lung cancer, mean age of death, standard deviation (SD) and 95% confidence interval of each time period. *p*-value refers to the Welch-ANOVA of male and female lung cancer patients.

**Table 2:** Multiple Comparisons.

	(a) Period	(b) Period	Mean difference (a − b)	*p*-value	95% CI
Male	1992–2001	2002–2011	−0.88	<0.001	−1.15; −0.61
		2012–2021	−2.60	<0.001	−3.14; −2.06
	2002–2011	1992–2001	0.88	<0.001	0.61; 1.15
		2012–2021	−1.72	<0.001	−2.28; −1.16
	2012–2021	1992–2001	2.60	<0.001	2.06; 3.14
		2002–2011	1.72	<0.001	1.16; 2.28
Female	1992–2001	2002–2011	0.50	0.02	0.07; 0.93
		2012–2021	−0.23	0.68	−0.94; 0.48
	2002–2011	1992–2001	−0.50	0.02	−0.93; −0.07
		2012–2021	−0.73	0.04	−1.42; −0.04
	2012–2021	1992–2001	0.23	0.68	−0.48; 0.94
		2002–2011	0.73	0.04	0.04; 1.42

Games-Howell *post-hoc* test.

Mean age of death of male lung cancer patient increased consistently throughout the observed time periods. As shown by Table 2, significant differences of mean values can be observed between each group. Overall, from the period 1992–2001 to the period 2012–2021, mean age of death increased sharply by 2.6 years, finally reaching the level of female patients.

**Figure 2:**
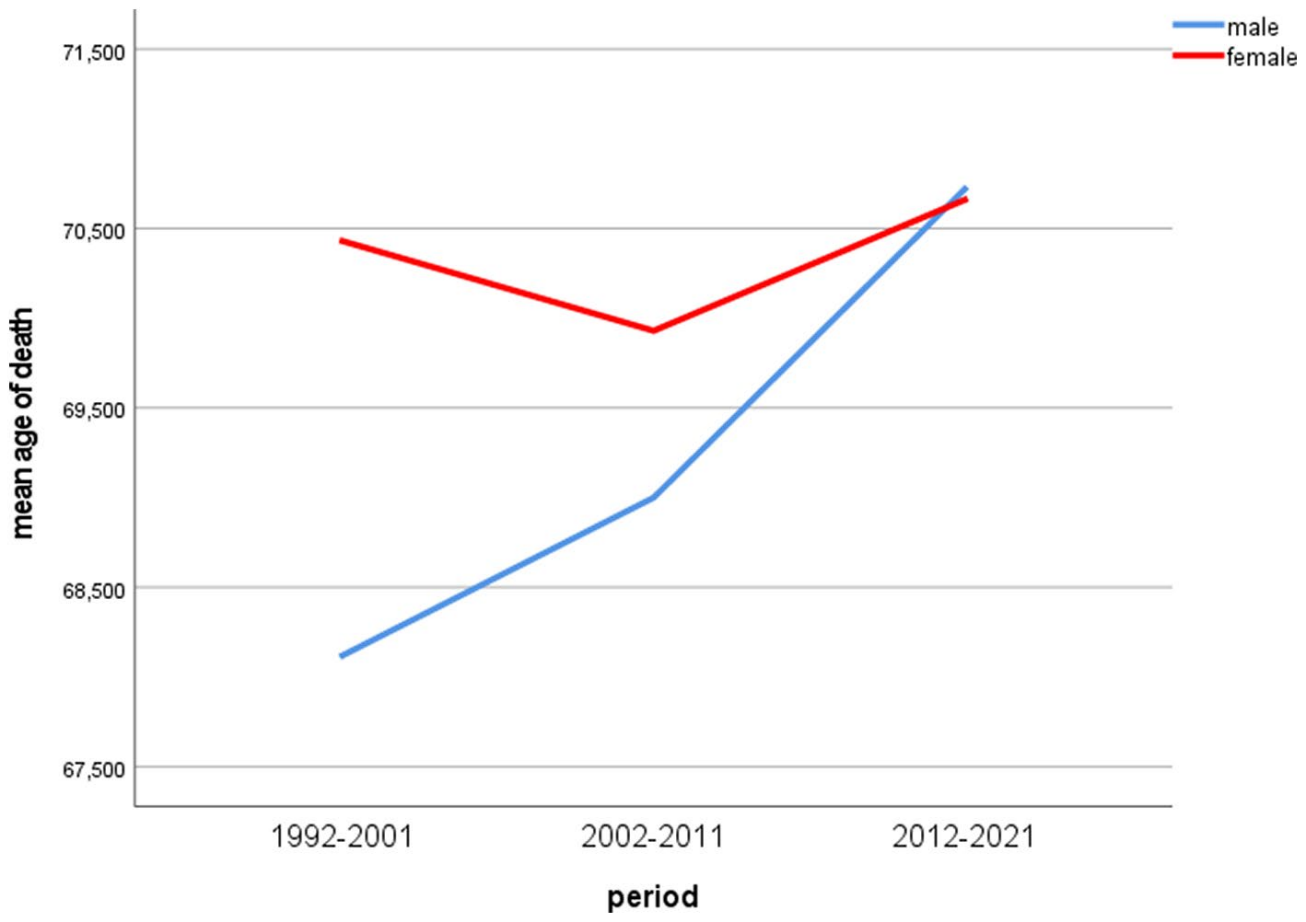
Mean age of death of male and female lung cancer patients 1992–2021.

Figure 2 shows the development of male and female mean age of death over the included time periods.

Differences of mean age of death between men and women were statistically significant in the periods 1992–2001 and 2002–2011 (*p* < 0.001) and accounted for 2.31 and 0.93 years, respectively. In the last period (2012–2021), the mean age of death of male lung cancer patients was 0.06 years higher than that of women, but the difference was statistically not significant in the independent samples *t*-test (two-tailed *p* = 0.85).

## 4. Discussion

The mean age of death provides substantial information, reflecting the length and intensity of exposure to risk factors as well as the quality of the health system (6). Nevertheless, epidemiologic and demographic studies rarely use the mean age of death as the core metric (10). This study reports the continuous increase of mean age of death of Austrian male lung cancer patients from the period 1992–2001 (68.12 years) to the period 2012–2021 (70.72 years; *p* < 0.001). In contrast, women showed no significant change between the first and the last period (70.43 and 70.66 years; *p* = 0.68). However, a small but statistically significant decline in the period 2002–2011 can be detected (69.93 years). The empirical mean age of death does not consider demographic changes in the observed population, especially changing age structures. Between 1990 and 2019, the life expectancy at birth of Austrian men and women increased by 7.3 years and 5.3 years, respectively. The remaining life expectancy at the age of 65 increased by 4.2 and 3.7 years for men and women in the same period (11). This demographic development in Austria might be partly reflected by the trend of mean age of death from lung cancer in men. However, the trend in women seems to be unaffected, as the mean age of death from lung cancer did not change significantly in the observed period.

As the reason for this development of mean age from lung cancer remains unclear, some possible influencing factors will be discussed in the following section.

In Western countries, patterns of smoking behavior have changed over time in women and men. International literature suggests that gender differences in smoking behavior still exist, but they are remarkably smaller in younger age groups (12, 13). In Austria, the share of men aged 16 years and above, who smoke on a daily basis, decreased continuously from 38.7% in 1972 to 23.7% in 2019, whereas the share of daily female smokers increased from 9.8% in 1972 to 22.2% in 2014 and decreased to 17.9% in 2019 (14). These changes in smoking prevalence are held responsible for the opposite trends of lung cancer incidence and mortality in Austria (6). Gender differences relating to the age of onset of regular smoking decreased during the past decades. In recent years, the age of smoking initiation of female smokers approached that of male smokers. According to data of the last Austrian Health Interview Survey (ATHIS 2019), 77.0% of male and 75.4% of female daily smokers aged 15–29 years started to smoke before the age of 17, whereas in the age group 45–59 years 62.0% of male and only 55.7% of female smokers initiated their smoking habit before the age of 17. Even bigger differences were detected in the higher age groups (14). Moreover, smoking intensity decreased in male as well as in female smokers since the early 2000s. In 2019, women still smoked less cigarettes per day than men (17.1 and 13.3 cigarettes). Regarding the share of heavy smokers, a decline can be observed across all three ATHIS waves. In 2006, about 20% of male and 8% of female smokers consumed more than 20 cigarettes per day. The percentage rates decreased in 2014 to 17.3 and 5.6% and in 2019 to 15.8 and 5.5%, respectively. Finally, when looking at the quit rates of Austrian smokers in 2019, the percentage of former smokers, relating to the (previously) smoking population, did not differ substantially between men and women (58.0% and 57.1%). Nevertheless, younger female smokers and accordingly women of reproductive age showed higher quit rates compared to men of the same age. In contrast, higher quit rates were observed in men aged 45 years and above. Overall quit rates of men and women increased in the last years from 48.0 and 45.0% in 2006, to 54.6 and 51.3 in 2014 and to 58.0 and 57.1% in 2019 (14–16).

To summarize, in recent years, male smoking habits took a turn to the better regarding various aspects like smoking prevalence, intensity and quitting. Although women show similar improvements in some smoking behaviors, they change for the worse in other aspects, especially smoking prevalence and age of smoking initiation. Remarkably, only minor differences in the reported age of smoking onset between young men and women were detected in the most recent ATHIS wave, whereas the gender gap was well defined in older age groups. This trend is observable not only in Austria, but also in other Western populations (12). Hence, it can be hypothesized that male smokers and lung cancer patients, respectively, benefit from recent advancements in early diagnosis and treatment of lung cancer with concomitant improvements of smoking habits, leading to a considerable increase of mean age of death from lung cancer. Contrariwise, women cannot take any further advantage of these clinical advancements, since early age of smoking onset is supposed to be an independent risk factor for the development of lung cancer. Studies suggest that this increase in risk continues until the age of 25 for women, but only until the age of 20 for men (17).

Moreover, it has to be mentioned that tar and nicotine yields of cigarettes decreased steadily since the 1950s, mainly due to improvements in filter technology and changes in cigarette design and composition. In the European Union, tar yields of cigarettes were reduced stepwise from 15 mg per cigarette as from 1993 to 12 mg as from 1998 and are now limited at 10 mg per cigarette since 2004 (18–20). Multiple studies demonstrated a dose-response relationship between tar exposure and lung cancer risk in cigarette smokers (21–23). Presumably, Austrian male smokers with their above-described smoking habits could benefit from these constant reductions of tar yields in cigarettes in terms of mean age of death. The increased usage of nicotine replacement therapy (NRT) might have further contributed in reducing tar exposure, especially in men, as supposed by several studies (24).

To conclude, the continuous increase of life expectancy at birth as well as of further life expectancy at the age of 65 in the last decades of the Austrian population is not reflected in female lung cancer patients and only slightly reflected in male patients. Thus, from a Public Health perspective, measures should increasingly focus on smoking behaviors of female adolescents and should endeavor to continue the recently observed decline of smoking prevalence among women in Austria. Particularly, price policy and taxation are effective strategies to reduce tobacco consumption and especially to prevent smoking initiation among adolescents (25, 26).

## Data availability statement

The original contributions presented in the study are included in the article/Supplementary material, further inquiries can be directed to the corresponding author.

## Author contributions

RF: conception of the work, data collection, data analysis, data interpretation, and drafting the article. UK: critical revision of the article and final approval of the version to be published. EG: data interpretation, critical revision of the article, and final approval of the version to be published. All authors contributed to the article and approved the submitted version.

## Funding

Article Processing Fee will be covered by the Medical University of Vienna.

## Conflict of interest

The authors declare that the research was conducted in the absence of any commercial or financial relationships that could be construed as a potential conflict of interest.

## Publisher’s note

All claims expressed in this article are solely those of the authors and do not necessarily represent those of their affiliated organizations, or those of the publisher, the editors and the reviewers. Any product that may be evaluated in this article, or claim that may be made by its manufacturer, is not guaranteed or endorsed by the publisher.
