# Supplementary figures and images for: Gender differences in lung cancer epidemiology – do Austrian male lung cancer patients still die earlier in life?
Source: Front Public Health. 2023 Apr 25;11:1099165. doi: 10.3389/fpubh.2023.1099165 (PMC10167001; doi:10.3389/fpubh.2023.1099165)

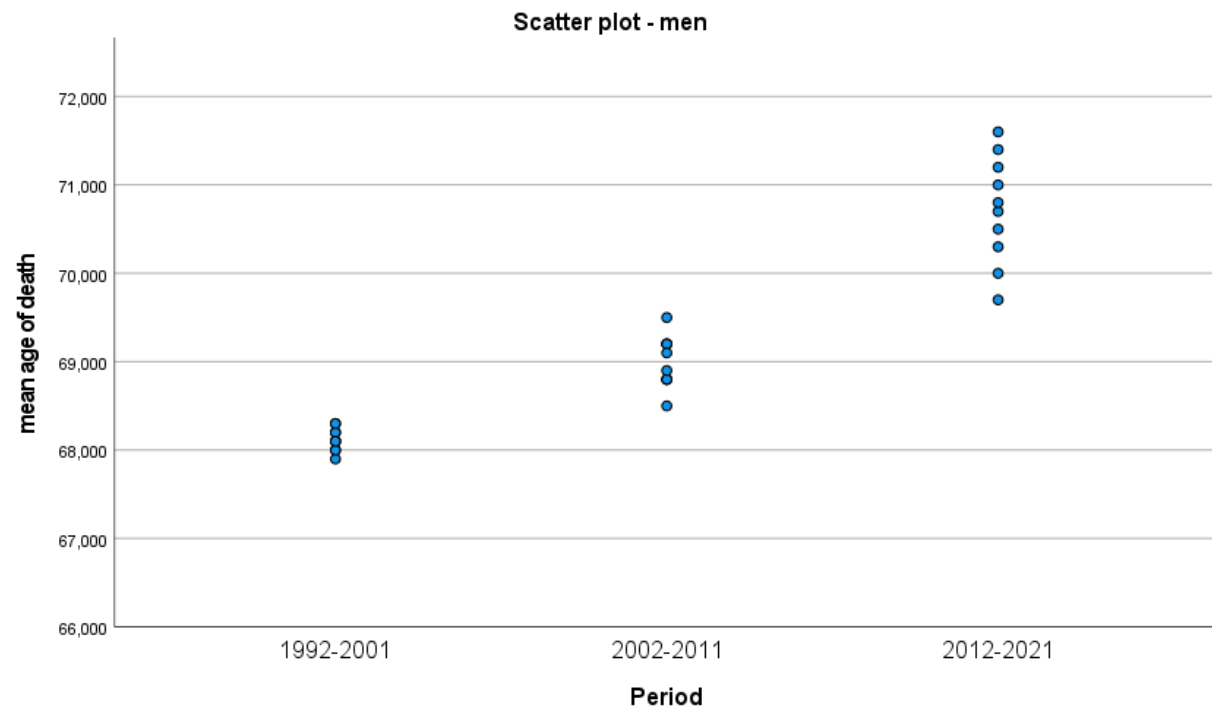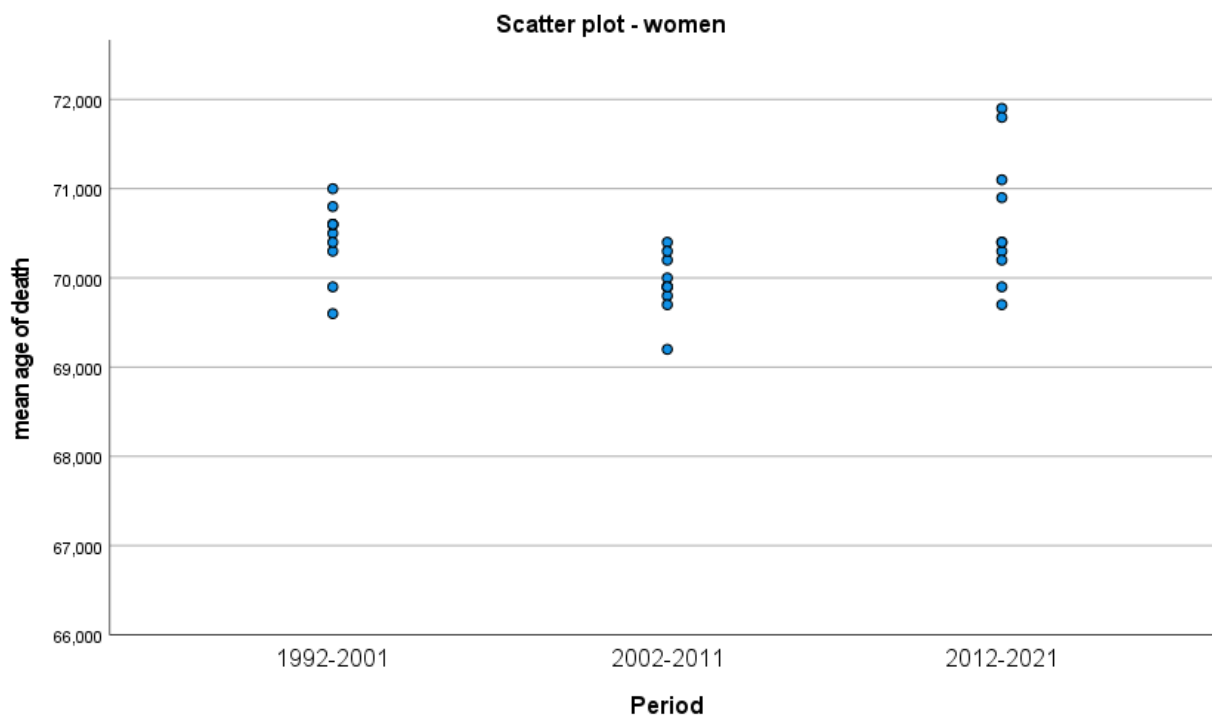

Supplement: Supplementary file 1 [file Image_1.pdf]
